# Rehabilitation Induced Neural Plasticity after Acquired Brain Injury

**DOI:** 10.1155/2018/6565418

**Published:** 2018-05-10

**Authors:** Andrea Turolla, Annalena Venneri, Dario Farina, Annachiara Cagnin, Vincent C. K. Cheung

**Affiliations:** ^1^Laboratory of Neurorehabilitation Technologies, IRCCS San Camillo Hospital Foundation, Venice, Italy; ^2^Department of Neuroscience, University of Sheffield, Sheffield, UK; ^3^Faculty of Engineering, Department of Bioengineering, Imperial College London, London, UK; ^4^Department of Neuroscience, University of Padova, Padua, Italy; ^5^School of Biomedical Sciences and KIZ-CUHK Joint Laboratory of Bioresources and Molecular Research of Common Diseases, The Chinese University of Hong Kong, Shatin NT, Hong Kong; ^6^Gerald Choa Neuroscience Centre, Brain and Mind Institute and Department of Biomedical Engineering, The Chinese University of Hong Kong, Shatin NT, Hong Kong

Motor skill learning refers to the process of optimizing sequences of action for accomplishing specific tasks [1]. Several mechanisms—genetic, neuroanatomical, and neurophysiological—are involved in this process, and they are mostly poorly understood. It has been suggested that the development of sequences of skilled movement involves the strengthening of the spatiotemporal relations between specific neuronal networks while weakening others. This process may occur via changes in connectivity between sets of corticospinal neurons following changes in synaptic efficacy [2]. The spatial and temporal organisation of synaptic weights is known as the “connectivity map”, whose existence has been postulated to be the biological substrate for functional behaviours. Some principles governing the organisation of connectivity maps in functional systems include the following: (1) the representation of any individual skill is highly distributed across different cortical regions (fractured somatotopy); (2) adjacent cortical areas are densely interconnected via white matter bundles (interconnectivity); and (3) the more demanding the skill, the larger the proportion of the map is involved in the skill's representation (area equals dexterity).

In recent years, new fundamental properties related to plasticity of connectivity maps have been revealed. Starting from evidence in the motor system [3, 4], it has been widely accepted that, following brain structural damage, both connectivity maps and behavioural skills can at least be partially restored through intense practice and rehabilitation [5]. These findings strengthen the idea that motor maps reflect a level of synaptic connectivity within the cortex that is required for the performance of skills. At the biological level, this idea was further confirmed by experiments on rats in which cholinergic inputs to the motor cortex were removed before skill training, thus preventing learning-dependent motor cortical map reorganization, with consequent impairment of motor learning [6]. On these grounds, skill training might induce plastic changes in synaptic efficacy within the motor cortex, with consequent changes in map topography. At the behavioural level, the most important parameter of task-oriented practice to induce brain plasticity is the intensity of training, defined as the amount of repetition executed for specific tasks. To induce an effective brain reorganisation, a certain threshold of training (i.e., minimum number of repetitions) needs to be reached. This effect is known as experience-dependent neuroplasticity. In animal models, it was estimated that between 1000 and 10,000 repetitions of the same task (trials) are needed before a permanent change at the synaptic level can be observed [7].

In animal experiments, it has been observed that behavioural motor learning is mediated by promotion of long-term potentiation (LTP) and by inhibition of long-term depression (LTD). None of these two mechanisms occurs with passive repetition without learning. The number of synapses and connections increases during the early stages of training [7], and in experimental settings, this phenomenon can be manipulated by injection of inhibitors of protein synthesis into the cortex, thus promoting or inhibiting skill learning [8]. Conversely, providing training sessions without learning might have detrimental effects such as a decrease in the number of synapses, weakened postsynaptic responses, and impairments in behavioural skills.

The possibility to induce functional reorganisation of cortical circuitries following lesions of the central nervous system through experience-dependent neuroplasticity has provided new perspectives in rehabilitation medicine, whose ultimate goal is to restore functions essential to independence in daily activities. *Neural Plasticity* sets out to publish a special issue devoted to the topic of *Rehabilitation Induced Neural Plasticity after Acquired Brain Injury*. The result is a collection of twelve outstanding articles submitted by investigators from 10 countries across Asia, Europe, North America, and Australia.

Current theories on the mechanisms underpinning recovery or compensation after brain injury have been outstandingly reviewed by M. J. Hylin et al. from the United States. Both human and animal models were considered to define how true recovery may be distinguished from compensation in the clinic. In this regard, the concepts of neuroanatomical effects of brain lesions, behavioural effects of recovery and compensation, brain functional reserve, and the impact of the timing and intensity of rehabilitation on neural plasticity were explored.

Two other reviews further provide a comprehensive background on rehabilitation-induced neural plasticity. M. Gandolfi et al. from Italy summarised the current available evidence on biomarkers mediating training-dependent recovery after stroke. Five groups of biomarkers were recognised as crucial for recovery: myokines, neurotrophic factors, neuropeptides, growth factors (GF) and GF-like molecules, and cytokines. On the other hand, more aiming at clinical translational research, L. Zhang et al. from China and the United States conducted a meta-analysis of the effects of low-frequency repetitive transcranial magnetic stimulation (LF-rTMS) on recovery of upper limb motor function and neuromodulation of cortical plasticity, after stroke. Overall, 22 studies were pooled for a total of 619 participants enrolled. Results indicated that stimulating the contralesional hemisphere with 1 Hz frequency has short-term efficacy on finger flexibility and activity dexterity. The above effects were also detectable as neurophysiological phenomena at the levels of resting-state motor threshold and motor-evoked potential. These authors concluded that LF-rTMS should be suggested as an add-on treatment to improve upper limb motor function in stroke survivors.

In addition to the aforementioned reviews and meta-analyses, three papers investigate specific mechanisms of rehabilitation-induced neural plasticity in rat models. M. Heredia et al. from Spain explored whether administering growth hormone (GH) followed by rehabilitation after severe injury of the motor cortex could have positive short- and/or long-term effects on the recovery of the affected upper limb. Results indicated that coupling GH with motor rehabilitation has positive effects when applied either immediately after or long after injury (i.e., 35 days postinjury). In contrast, GH administration alone resulted in improved nestin and actin reexpression, but without significant changes at the behavioural level. H. J. Kim et al. explored the use of anodal transcranial direct current stimulation (tDCS) to promote recovery of motor and somatosensory functions after repetitive mild traumatic brain injury (rmTBI). For both motor-evoked potential (MEP) and somatosensory-evoked potential (SEP), amplitude and latency were larger in the tDCS group but shorter in the sham-tDCS group. These results suggest that anodal tDCS might be a useful tool for promoting transient motor recovery by increasing synchronization of cortical firing, even in human survivors soon after concussions. C. Liu et al. from China explored the effect of acoustic trauma on the central and peripheral nervous systems. Changes in parvalbumin-containing neurons (PV neurons) were investigated in rats exposed to a one-hour noise stimulation. Results indicated that PV neurons were more apparent in the cortex of the noise-exposed group, suggesting the presence of a compensatory mechanism that maintains a stable state of the brain.

Four original clinical trials offer new evidence on the neuroplastic effects conferred by established rehabilitation modalities (i.e., prismatic adaptation, tDCS, and robotics) for stroke survivors. I. Tissieres et al. from Switzerland tested whether rightward prismatic adaptation (R-PA) might reduce visuospatial neglect symptoms. Results in stroke survivors with unilateral spatial neglect showed that in the half of tested subjects, extinction of dichotic listening in the left ear was alleviated by R-PA. Interestingly, the brain lesions of the nonresponders all involved the right dorsal attentional system and posterior temporal cortex. These authors concluded that shifting in hemispheric dominance within the ventral attentional system might be an appropriate model for interpreting these behavioural results. G. Pellegrino et al. from Italy tested, using magnetoencephalography (MEG), whether neural plasticity is induced by bilateral tDCS in the sensorimotor areas. They conducted a double-blind randomized controlled trial where tDCS was compared with sham-tDCS. Results indicated that tDCS reduced left frontal alpha, beta, and gamma power while global connectivity in delta, alpha, beta, and gamma frequencies increased. M. Gandolfi et al. from Italy and Austria quantified the neural electroencephalography (EEG) correlates of robot-aided bilateral arm training (BAT) for the recovery of upper limb function after stroke. Results indicated that providing robot-aided BAT induced complex shifting of desynchronization across hemispheres in a task specific manner. Specifically, alpha and beta desynchronization that were bilateral in the sensorimotor areas before training became, after training, ipsilesional to the stroke-affected hemisphere for passive movements of the affected hand and contralesional during active movements of the affected hand. These authors concluded that robot-aided BAT might be helpful to promote differentiation of EEG patterns after stroke. F. Capone et al. from Italy designed a proof-of-concept study to explore the effect of combining robotic rehabilitation with noninvasive vagus nerve stimulation (VNF) for the recovery of upper limb motor function after stroke. The study was a double-blind, semi-randomised, and sham-controlled trial involving 14 patients with diverse presentations. Their results demonstrate that this innovative modality was safe and that patients receiving robotics with VNS gained better motor functions after treatment than patients treated by sham VNS.

Finally, two longitudinal studies investigate neuroimaging biomarkers for ageing subjects and stroke survivors. M. De Marco et al. from the UK and Italy, by studying the effect of small white matter lesions accumulated in the ageing brain, suggested that even in the absence of overt disease, the brain responds with a compensatory (neuroplastic) response to the accumulation of white matter damage over time, thus leading to increases in regional grey matter density and modifications in functional connectivity. K. S. Hayward et al. from Canada and Australia explored whether structural brain biomarkers (e.g., functions of the corticospinal tract, transcallosal inhibition, and their own fractional anisotropy) can predict severity of motor impairment after stroke. Cluster analysis indicated that functionality and structure of the corpus callosum can predict which patients have chronic and severe motor impairment of upper limb motor function.

Overall, this collection of papers argues convincingly that for the rehabilitation field, now is the time to forge even more pipelines going from basic neuroscientific results to novel, effective interventions for patients [9]. Our knowledge in the neural plasticity induced by rehabilitation in people with acquired brain injuries has reached the critical point that demands any proposal of innovative approach to be founded on both hard scientific rationale and robust clinical evidence of effectiveness. More importantly, we believe that a more solid understanding of the neuroscientific basis underpinning the effectiveness of some promising existing interventions should not only allow their further improvement but also promote their appropriate use in current clinical practice and delivery of rehabilitation services. Hopefully, this publication and other recent studies will encourage more clinicians, rehabilitation engineers, and neuroscientists to work together to further dissect the neural mechanisms underscoring functional recovery after acquired brain injuries and translate these findings into novel therapies.
